# Effective maNagement of depression among patients witH cANCEr (ENHANCE): a protocol for a hybrid systematic review and network meta-analysis of randomised controlled trials of interventions for depressive symptoms

**DOI:** 10.1186/s13643-022-02107-y

**Published:** 2022-11-12

**Authors:** Maria M. Pertl, Sergio Perez, Sonya Collier, Emer Guinan, Garret Monahan, Katie Verling, Emma Wallace, Aisling Walsh, Frank Doyle

**Affiliations:** 1 Department of Health Psychology, School of Population Health, Royal College of Surgeons in Ireland (RCSI), 123 St. Stephen’s Green, Dublin, 2 Ireland; 2 School of Social Work and Social Policy, Trinity College Dublin (TCD), Dublin, 2 Ireland; 3 Psycho-Oncology Unit, St. James’s Hospital Dublin, Dublin, 8 Ireland; 4 Trinity Exercise Oncology Research Group, Discipline of Physiotherapy, Faculty of Health Sciences, TCD, Dublin, 2 Ireland; 5 ENHANCE PPI Panel, Dublin, Ireland; 6 Department of General Practice, , University College Cork, Cork, Ireland; 7 Department of Public Health and Epidemiology, School of Population Health, RCSI, 123 St. Stephen’s Green, Dublin, 2 Ireland

**Keywords:** Systematic review, Network meta-analysis, Depression, Cancer, Pharmacotherapy, Psychotherapy, Exercise, Collaborative care, Complementary and alternative medicine, Public and patient involvement

## Abstract

**Background:**

Depression is common among patients with cancer and is associated with lower treatment participation, lower satisfaction with care, poorer quality of life, greater symptom burden and higher healthcare costs. Various types of interventions (e.g. pharmacological, psychotherapy) are used for the treatment of depression. However, evidence for these among patients with cancer is limited. Furthermore, the relative effectiveness and acceptability of different approaches are unknown because a direct comparison between all available treatments has not been carried out. We will address this by conducting a network meta-analysis (NMA) of interventions for depression among people with cancer using a hybrid overview of reviews and systematic review methodology.

**Methods:**

We will search for and extract data from systematic reviews of randomised controlled trials (RCTs) of depression interventions for patients with cancer from inception, before performing a supplemental search for more recent RCTs. We will include RCTs comparing pharmacological, psychotherapy, exercise, combination therapy, collaborative care or complementary and alternative medicine interventions with pill placebo, no treatment, waitlist, treatment as usual or minimal treatment control groups, or directly in head-to-head trials, among adults who currently have cancer or have a history of any cancer and elevated depressive symptoms (scores above a cut-off on validated scales or meeting diagnostic criteria). Our primary outcomes will be change in depressive symptoms (standardised mean difference) and intervention acceptability (% who withdrew). Our secondary outcomes will be 6-month change in depressive symptoms, health-related quality of life, adverse events and mortality. We will independently screen for eligibility, extract data and assess risk of bias using the RoB 2 tool. We will use frequentist random-effects multivariate NMA in Stata, rankograms and surface under the cumulative ranking curves to synthesise evidence and obtain a ranking of intervention groups. We will explore heterogeneity and inconsistency using local and global measures and evaluate the credibility of results using the Confidence in NEtwork Meta-Analysis (CINeMA) framework.

**Discussion:**

Our findings will provide the best available evidence for managing depression among patients with cancer. Such information will help to inform clinical guidelines, evidence-based treatment decisions and future research by identifying gaps in the current literature.

**Systematic review registration:**

Submitted to PROSPERO (record number: 290145), awaiting registration.

**Supplementary Information:**

The online version contains supplementary material available at 10.1186/s13643-022-02107-y.

## Background

Patients with cancer have a fivefold increased odds of depressive symptoms compared to the general population, with one in four experiencing clinically significant symptoms [1]. Moreover, the risk of major depression in patients with cancer is two-to-three times the estimated prevalence in the general population [2]. Prevalence rates span from 16 to 25% [3], with estimates varying widely due to different methodological standards, the various screening methods and diagnostic instruments used and cancer type and severity [3–5]. Determining the presence of depression among patients with cancer is also particularly challenging because it can be difficult to distinguish between normal psychological distress and sadness in response to cancer and clinical depression [6]. In addition, symptoms associated with cancer and side effects associated with its treatment (e.g. fatigue, weight loss and sleep disturbance) overlap with the diagnostic criteria for depression [6]. Nevertheless, identifying and treating depression are of critical importance, not only for the welfare and quality of life of people with cancer but also for maximising the effectiveness, efficiency and acceptability of cancer treatments.

Depression among patients with cancer has been associated with lower treatment participation; lower satisfaction with care and quality of life; higher levels of anxiety, pain and fatigue; and higher healthcare costs, resource use and mortality [7–9]. Furthermore, even depressive symptoms in the absence of a formal diagnosis of major depression have been found to be an independent risk factor for mortality and disease-related burden (e.g. [9–12]). Therefore, identifying treatments for depression that are effective and acceptable to patients with cancer is of key clinical importance, and greater awareness of the best management strategies may also encourage health professionals to more routinely assess and treat depression in clinical practice [13].

Many different types of interventions are used, often in combination, for the treatment of depression among patients with cancer [14]. These include psychotherapies, such as cognitive behavioural therapy (CBT), problem-solving therapy, supportive therapy, counselling and group therapy; pharmacological interventions, including antidepressants and stimulants; exercise interventions; and collaborative care interventions, which involve active collaboration between various health professionals (e.g. medical doctors, case managers and mental health specialists) and the patient in managing the problem, as well as complementary and alternative medicine (CAM) interventions, such as acupuncture [14–19]. However, evidence for the effectiveness, cost-effectiveness and acceptability of these interventions among patients with cancer is limited and mixed [16, 18, 20]. As a result, implications for practice are unclear, and clinical guidelines have had to draw on data from depression interventions for the general population rather than cancer-specific studies [18, 20]. However, the benefits of and adverse effects associated with interventions may be different among patients with cancer and the general population [16]. Furthermore, since there is no evidence for the superiority of one intervention over another, guidelines have been limited to a large extent to broad statements on the general effectiveness of various approaches [19].

Obtaining information on the relative effectiveness and acceptability of different types of interventions for depression would enhance the provision of psycho-oncological care by providing guidance to clinicians around what type of interventions is most appropriate and providing policy-makers with excellent evidence for resource allocation. Such data are not available as direct comparisons between all available depression treatments have not been carried out; nevertheless, given the plethora of randomised controlled trials (RCTs) and meta-analyses that have evaluated specific interventions for depression among patients with cancer (e.g. [16, 21, 22]), there are sufficient data for combined direct and indirect comparisons of existing interventions using network meta-analysis (NMA).

NMA is an advanced technique that can fill gaps in the literature by making indirect comparisons between treatment options that have not consistently been made in head-to-head formats and can generate hierarchies of which treatments are the best, second best and so on in terms of outcomes of interest [23, 24]. For example, it has previously been used to compare the efficacy and acceptability of antidepressant drugs for the treatment of major depressive disorder [25]. Furthermore, it can compare interventions that are very different in nature, such as medication and exercise (e.g. [26, 27]), even when comparator groups may differ [27, 28]. Such crucial information is urgently needed by clinicians and policy-makers but cannot be provided using conventional meta-analysis. Indeed, there is growing recognition that systematic reviews based on NMA constitute the highest level of evidence in treatment guidelines [24]. We propose to conduct an NMA summarising the current literature on the treatment of depression among patients with cancer and ranking competing interventions in terms of efficacy and acceptability. A comparison of the efficacy of interventions for depression among patients with coronary artery disease has already been completed and has informed our methodological approach [27].

## Objectives

The main objective of this NMA is to compare the efficacy and acceptability of established treatments for managing depression among patients who have had a diagnosis of cancer. The primary research questions are as follows: (1) what treatments are the most effective in reducing depressive symptoms among patients with cancer, and (2) what treatments are the most acceptable for managing depressive symptoms among patients with cancer? Using the patient, intervention, comparison, and outcome (PICO) framing, our criteria to search for studies to answer these questions will be as follows:

- **Participants**: (a) Adult participants aged 18 years and over; (b) with cancer or a history of any cancer; (c) elevated depressive symptoms (i.e. scoring above the cut-off criteria on a validated depression screening instrument or meeting the diagnostic criteria on a standardised clinical diagnostic interview (e.g. the DSM-5 [29]) for assessing major depressive disorder, adjustment disorder or dysthymic disorder) at the time of study entry; and (d) enrolled in an RCT targeting depressive symptoms in any setting
- **Interventions**: Any established treatment for depression including pharmacotherapy, psychotherapy, exercise, combination therapy and collaborative care, as well as complementary and alternative medicine (CAM) interventions
- **Comparison**: Any appropriate comparator group including usual care, placebo groups, no intervention, waitlist, attention control group or another depression treatment
- **Outcomes**
  - ° **Primary**
    - Efficacy response (depressive symptom mean change scores between groups) after 8 weeks (range 4–12 weeks) from baseline on validated measures of depression
    - Acceptability (percentage of patients who discontinued with the intervention/comparator for any reason) at any stage
  - ° **Secondary**
    - Longer-term follow-up: depression assessed at 26 weeks (range 20 and 30 weeks) on validated measures of depression
    - Health-related quality of life (HRQoL) or, if this is unavailable, general QoL (mean change scores between groups) after 8 weeks (range 4–12 weeks) from baseline on validated scales. If sufficient data are available, we will subdivide QoL into physical, social and emotional domains.
    - Adverse events (the percentage of participants who leave the study as a result of intervention-related adverse events) occurring within 12 weeks of study commencement
    - Mortality (all cause; the percentage of participants who die after or during the treatment [cancer related or otherwise]) for the longest duration of follow-up

## Methods

This protocol closely follows the methods of Doyle et al. [28] and the PRISMA extension statement for the reporting of systematic reviews incorporating NMA [30]. We have provided a completed PRISMA-P checklist in Additional file 1. If any amendments to this protocol are necessary during the review process, we will add details and justifications for these to the registration record and report these in the final systematic review results report.

### Public and patient involvement (PPI)

PPI helped us to refine the focus of the research questions, and PPI will continue to play a key role in the running of this study. A PPI panel comprising of two experts by experience (GM and KV) was recruited to the steering committee at the outset of the project. They were given training on the study methods and worked with the other members of the research team to refine the PICO criteria for study eligibility. Going forward, the PPI panel will assist with the interpretation of NMA results, identifying implications for practice, and the dissemination of findings. We have reported PPI in the development of this protocol using the short form of the Guidance for Reporting Involvement of Patients and the Public 2 (GRIPP2-SF [31];), which is available in Additional file 2.

### Eligibility criteria

#### Study types

Eligible studies will be RCTs of depression interventions in patients who currently have cancer or have a history of any cancer. The interventions of interest, based on clinical guidelines for the management of depression among patients with cancer and/or chronic physical health problems [19, 20, 32, 33], are pharmacotherapy, psychotherapy, exercise, combination therapies and collaborative care interventions, as well as CAM approaches. As an outcome measure, studies using any of these interventions should employ a validated depression scale or diagnostic interview able to report a (potential) change in depression or depressive symptoms from baseline or pre- to post-treatment. However, depression does not necessarily need to be specified as the primary outcome; studies that report depression as one of a number of primary outcomes or a secondary outcome will still be eligible for inclusion provided that they meet the other inclusion criteria. Psychological interventions that are not established psychotherapies and are not delivered by professionally trained therapists will be excluded from the study. As recommended by Chaimani et al. [34], additional unspecified interventions that surface during the search process may be considered for inclusion in the network if the study meets the eligibility criteria and the inclusion of the intervention could serve to supplement the analysis by, for example, increasing the precision of the results. Studies included will be published (in English) in peer-reviewed journals, review articles or RCT registries.

#### Participants

Participants will be 18 years of age and over, and currently have cancer or have a history of any cancer, and be at any stage of treatment (pretreatment, active treatment, or post-treatment). Participants must be enrolled in an RCT targeting elevated depression as either a primary or secondary outcome, assessed using validated measures of depression symptoms at baseline and post-intervention. As thresholds for elevated depression may not always be reported, and data may not always be available from authors, we will include participants from trials that do not explicitly specify elevated depression as an inclusion criterion as long as the mean depression score at baseline of all intervention and control groups is at least one standard deviation above the clinical cut-off on a validated measure of depression. We will exclude participants if they have (a) a diagnosis other than cancer, (b) antenatal/postnatal depression, (c) bipolar disorder or psychotic depression, or concurrent secondary psychiatric diagnoses. If some, but not all, of a study’s participants are eligible for inclusion (e.g. if they include patients with cancer and patients with other diseases), then we will request the data for eligible patients only from the authors or, if > 80% of participants are eligible for the review, we will include the overall trial estimates.

#### Intervention types

We will include the following types of interventions; however, as recommended, unspecified interventions may also be included post hoc to improve the precision of the model [34].

##### Pharmacotherapy

Interventions in this category will comprise of any licensed medicines used to treat the symptoms of depression. In assessing eligibility, we will draw on clinical guidelines (e.g. [19, 20, 32]), being mindful of changes in recommended treatments over time. Examples include selective serotonin re-uptake inhibitors (SSRIs), serotonin-norepinephrine reuptake inhibitors (SNRIs), tricyclic anti-depressants, monoamine oxidase inhibitors, mirtazapine and agomelatine. We will only include studies that randomised participants to pharmacotherapies within their licensed dose range [35].

##### Psychotherapy

We will adopt the inclusion criteria used by Cuijpers et al. [36] for the development of their complete database of trials on psychological treatments for depression (www.evidencebasedpsychotherapies.org). These are based on the definition of psychotherapy by Norcross [37]: “Psychotherapy is the informed and intentional application of clinical methods and interpersonal stances derived from established psychological principles for the purpose of assisting people to modify their behaviours, cognitions, emotions, and/or other personal characteristics in directions that the participants deem desirable”. Eligible interventions can be delivered by any therapist (including psychologists, nurses, social workers) so long as they are trained to deliver the therapy and in any treatment format so long as they are facilitated (i.e. individual, group, telephone, guided self-help or couple therapy). As outlined in Cuijpers et al. [36], these fall into the following eight psychotherapy categories: (1) cognitive behaviour therapy (CBT), (2) behavioural activation therapy, (3) problem-solving therapy, (4) interpersonal psychotherapy, (5) psychodynamic therapy, (6) nondirective therapy, (7) third-wave therapies (e.g. acceptance and commitment therapy, mindfulness-based stress reduction, mindfulness-based cognitive therapy) and (8) life review therapy (for definitions and examples, see https://evidencebasedpsychotherapies.shinyapps.io/metapsy/_w_ed60cf71/variable_description.pdf).

##### Combination therapy

Interventions in this category will involve both psychotherapy and pharmacotherapy components.

##### Exercise interventions

We will include interventions that involve aerobic and/or resistance training exercise that is “prescribed” according to evidence-based FITT principles, which define the frequency, the intensity, the type and the timing of the exercise programme being prescribed. Use of the FITT principles is important because it provides clarity regarding the volume and nature of exercise that the intervention entails, enables adjustments or modifications to the exercise prescription to be accurately captured and thereby facilitates transparency on the actual dose that is received. This is critical for the replication of interventions and application into “real-world” settings as greater or lesser volumes of exercise may lead to greater or lesser effects [33, 38]. Other types of exercise, such as yoga or Tai Chi, which are not delivered based on the prescription principles for exercise training, will not be included in this intervention group [33, 39, 40]. While the potential value of such forms of exercise is recognised, it is difficult to generate definitive prescriptions for these types of exercise as they often include non-exercise components [33]. However, we will include them as part of the complementary and alternative medicine group, as appropriate, as specified below.

##### Collaborative care interventions

Interventions in this category will involve a multicomponent approach with active collaboration and enhanced inter-professional communication between different specialists and primary care providers [41]. As such, we will include trials on interventions that consider both pharmacologic and psychological treatment options for patients within the context of an integrated management team that includes a patient care manager, a psychiatrist and an oncologist or primary care provider [42, 43].

##### Complementary and alternative medicine (CAM)

CAM interventions involve therapeutic approaches that are not usually included in conventional Western medicine [44], and they are used by patients in combination with or as alternative treatments for depression [45]. In line with the National Centre for Complementary and Integrative Health and van der Watt et al. [46], we will include approaches covered in the following classifications: herbal interventions, nutritional supplements (e.g. vitamins or probiotics) and aromatherapy; cognitive interventions (e.g. hypnotherapy, imagery, and meditation); and physical interventions (e.g. Tai Chi, acupuncture and light therapy).

#### Comparison groups

To qualify for inclusion, RCTs must compare interventions with another appropriate comparator group such as treatment as usual (TAU), enhanced usual care, pill placebo control groups, no treatment, waitlist, attention control groups or another depression treatment. Comparator groups will be considered separately as previous work has shown that these are not equivalent [27, 47, 48]. The nature of the control groups used can, for example, have a major influence on the results by impacting on risk of bias (such as attrition rates and blinding), heterogeneity and effect sizes observed [47, 48]. With this in mind, we will categorise comparators into the following three groups in line with recommendations [49] and previous research [27, 28]:

1. Pill placebo (for drug trials)
2. No treatment, waitlist or treatment as usual
3. Treatment control (defined as minimal treatment control, active comparator and specific and non-specific factors treatment control)

We will contact authors for further information in instances where comparator groups are unclear and, if necessary, include an “unclear” comparator group or exclude a study from the NMA if details on comparator groups are not available.

**Fig. 1:**
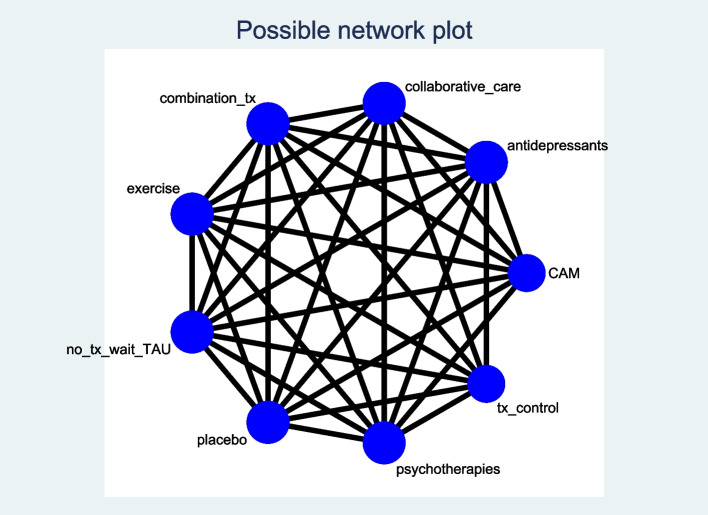
Sample network of all possible depression intervention and control group comparisons

Figure 1 below shows a sample network plot, based on all of the possible depression interventions and comparison groups we plan to include.

#### Outcomes

##### Primary outcomes

We will include two primary outcomes, following the example of Doyle et al. [28] and Cipriani et al. [25]:

1. Efficacy: Depression (means and standard deviations [SDs]) measured using validated tools (diagnostic interviews or screening instruments) and summarised with standardised mean difference (SMD) from baseline to post-intervention. The follow-up measure closest to 8 weeks will be used; however, measures within a range of 4–12 weeks will be accepted. If multiple depression measures were included in a given study, scores on the Hamilton Depression Rating Scale (HAM-D) will be used; if the HAM-D is not included, then preference will be given to longer scales with better content validity [35, 50].
2. Acceptability: The percentage of participants who discontinue the intervention/comparator for any reason, at any stage

##### Secondary outcomes

The secondary outcomes of interest are as follows:

1. Longer-term follow-up efficacy: Depression (means and SDs) measured using validated tools (diagnostic interviews or screening instruments) and summarised with SMD from baseline to follow-up (the measure closest to 26 weeks available will be used, between 20 and 30 weeks).
2. HRQoL: HRQoL scores (means and SDs) on physical, social and emotional domains summarised using SMD from baseline to post-intervention. As for the primary depression outcome, the measure closest to 8 weeks will be used with an acceptable range from 4 to 12 weeks. Generic QoL scores will be used when HRQoL scores are not available.
3. Adverse events: The percentage of participants who experience intervention-related adverse effects within 12 weeks of study commencement
4. Mortality (all cause): The percentage of participants who die after or during the treatment (cancer related or otherwise) for the longest duration of follow-up

### Search strategy and study selection

As numerous systematic reviews exist on this topic (e.g. [16, 21, 22, 51]), we will carry out a hybrid review of reviews and systematic review methodology [27]. This approach involves first searching for relevant systematic reviews and extracting the pertinent references from these, before performing a supplemental search for individual RCTs that were published more recently (e.g. within the last 5 years, depending on the dates of the systematic reviews). By drawing on the work of previous systematic reviewers, this approach is less resource intensive, saving time and effort, while still covering the available literature [27, 52]. We will use the following databases to search from inception for reviews and meta-analyses: Cochrane Library, CINAHL, MEDLINE/PubMed, MEDLINE In-Process, Embase and PsycINFO. We will independently screen the search results for eligible systematic reviews by applying the same inclusion criteria outlined in the previous section, whereby the systematic reviews will only need to include at least some studies that match the trial eligibility criteria. We will extract eligible RCTs and their associated data from the collected reviews and original studies. In addition, we will perform an updated search for relevant RCTs. We anticipate that the time-period for these searches will be within the last 3–5 years; however, the range will be determined by how recent the available systematic reviews are. For RCT searches, we will use the databases MEDLINE/PubMed, PsycINFO, and the Cochrane Library and clinical trials registries [53], which includes RCTs indexed in PubMed, Embase, and CINAHL as well as the ClinicalTrials.gov and the International Clinical Trials Registry Platform (ICTRP) clinical trial registries. Furthermore, we will also search the reference lists of all included RCTs. Searches will adopt the BMJ trials and SR filters (available at: https://bestpractice.bmj.com/info/toolkit/learn-ebm/study-design-search-filters/). Although searches will not be filtered by language, only English language articles will be included. We have provided a sample search strategy in Additional file 3. We will download references into the reference manager software EndNote and remove duplicate references via the software tools. Two reviewers will independently select reviews and trials and review full texts for inclusion based on the eligibility criteria outlined in the previous section, discussing disagreements with a third reviewer.

### Data extraction

Data extraction will be completed using structured data extraction spreadsheets in Excel to obtain all relevant data in a consistent fashion. Double data entry will be carried out, whereby data will be inputted independently by two reviewers into two separate datasets and then compared. Details extracted from the data will include study characteristics (first author, year of publication, journal, setting and country), participant characteristics (sample size, mean age, % female, type of cancer, cancer stage [i.e. early-stage disease vs. advanced stage/palliative care]), time since diagnosis, cancer treatment stage [i.e. pretreatment, active treatment, post-treatment], depression inclusion criteria, baseline depression severity, depression assessment tools, the presence of premorbid depression) and intervention and comparator group details (type of pharmacotherapy [name, dose, duration]; for psychotherapy interventions, the Template for Intervention Description and Replication checklist [TIDiER] [54] will be used for extraction of headings (i.e. intervention name, rationale/theory, materials, procedures, who delivered the intervention, delivery mode, location/setting, dose/intensity, tailoring, modifications, fidelity). Since psychotherapies can take many modalities and are delivered in different forms, the TIDiER headings will allow more precise documentation of any significant disparities among the selected psychotherapeutic treatments selected for study. Data extracted from the original RCT reports, including multi-arm trials, will be used to calculate summary effect sizes.

### Continuous outcomes

For continuous outcomes, we will extract SMDs (when reported), 95% confidence intervals, means, SDs and number of patients participating in trial arm of the study into the final dataset. If this information is not available, we will request these data from the RCT authors. If data are omitted in the reports (e.g. SDs not reported), we will impute using the Cochrane recommended techniques for estimating SDs [55] and the *metaeff* command procedure in Stata to calculate SMDs and 95% confidence intervals from available data [56]. If mean change scores are not reported, then we will consider outcome scores [27]. If trials report the percentage of participants who no longer have elevated depression following the intervention, rather than mean change scores or outcome scores, we will also use the *metaeff* command to calculate SMD. If sufficient data are not available to calculate the SMD, we will include the study for descriptive purposes only and exclude it from the main NMA. If insufficient data are available to calculate the 95% confidence intervals, we will consider imputing based on the median from the other studies from that particular group [27, 28]. We will carry out sensitivity analyses to determine whether there are implications of such imputations [27].

### Binary outcomes

For the extraction of binary outcomes (i.e. acceptability, mortality, adverse events), we will obtain the number of participants with each event from each trial arm. When data are not available, we will contact the authors of the studies to request the information.

### Duration of RCTs and outcome assessments

Following previous methods [25, 27, 28, 35], we will adopt an 8-week threshold for the synthesis of the primary depression outcome and the secondary QoL outcome. In those cases where data are unavailable for that duration, the closest available data from 4 to 12 weeks will be used [25]. We will use overall dropout rate, regardless of time-point as the second primary outcome for acceptability. We will use long-term depression assessments at 26 weeks (with an acceptable range between 20 and 30 weeks) as a secondary outcome.

### Missing RCT outcome data and units of analysis

We will extract all data as they were reported in the original trials, regardless of how missing data were dealt with in each study. As part of our risk assessment, we will rate whether or not the handling of missing was appropriate or not, where possible drawing this information from previous systematic reviews [27, 57]. In line with previous NMAs [25, 28, 35], we will extract pertinent data to explain clustering from cluster randomised trials and extract only data relating to the first study period from cross-over trials to prevent carry-over effects.

### Risk of bias and quality of evidence

Two reviewers will independently extract and assess risk of bias (RoB) data, which will be used to inform updated RoB 2 tool [58] ratings. Of note, our use of the RoB 2 tool is likely to lead to fewer studies being classed as having a high RoB than existing systematic reviews that used the Cochrane tool [55], in particular for trials for which it was not possible to blind for treatment assignment. This is likely because the RoB 2 involves a more nuanced decision-making framework that does not automatically consider unblinded studies to be at high risk of bias [58]. For example, for trials in which blinding was not feasible or implemented, the RoB 2 considers whether post-randomisation deviations from the intervention led to bias or whether the reasons for missing outcome data contributed to bias; if not, such trials can still meet the criteria for low RoB. If data to assess risk of bias are insufficient or missing, we will consider contacting RCT authors to obtain additional information. In instances when the two independent reviewers disagree on RoB ratings, input from a third reviewer will help to settle disagreements.

We will use the Confidence in Network Meta-Analysis (CINeMA) framework to evaluate the credibility of our results [59] and present the resulting information in a summary table. CINeMA is based in part on the GRADE (Grading of Recommendations Assessment, Development and Evaluation) framework [60] but has been specifically developed to account for the complexity of NMA methods. The framework considers six domains: (i) within-study bias, (ii) reporting bias, (iii) indirectness, (iv) imprecision, (v) heterogeneity and (vi) incoherence; and, as with GRADE, assessments of each domain are summarised to reflect whether confidence for the treatment effect is very low, low, moderate or high [59]. We will use CINeMA to summarise the strength of the evidence for each of the primary outcomes from the network estimates.

### Transitivity assessment

Transitivity relates to how effect modifiers are distributed across intervention comparisons [23]. To uphold this key assumption of NMA, studies making different direct comparisons must be sufficiently similar in all respects other than the intervention that is being examined [61], such that it is valid to make indirect comparisons between intervention groups that are connected via one or more intermediate comparator groups [34]. Previous NMAs on the efficacy of antidepressants have identified that factors such as bipolarity, psychotic features and subthreshold depression can moderate the efficacy of antidepressants and therefore, to uphold the transitivity assumption of the network, have limited samples to nonpsychotic patients with unipolar depression [35]. We will take a similar approach and, in line with Doyle et al. [28], exclude studies where 20% or more of participants have bipolar or psychotic depression or concurrent secondary psychiatric diagnoses. Another factor that may moderate the efficacy of interventions for depression is whether participants enrolled in an RCT have elevated depressive symptoms (i.e. score above a specified threshold on a validated measure of depression) at baseline. Including trials that do not specify elevated depression as an inclusion criteria may misrepresent the efficacy of interventions, because participants who have subthreshold levels of depression to begin with have less scope to improve on this outcome [21]. Indeed, previous studies have demonstrated that baseline severity of depression moderates the efficacy of psychosocial treatments for patients with cancer, such that effects are negligible when baseline depression is low [62]. Therefore, we have specified that only RCTs that specifically enrolled participants with elevated depressive symptoms meet our inclusion criteria. Given these precautions, we assume that participants who fulfil the inclusion criteria for this protocol are equally eligible to be randomised to any of the intervention groups. Nevertheless, to assess transitivity and further guard against violating this assumption, we will compile a list of potential effect modifiers from data collected (e.g. participant age, sex, cancer type, treatment stage, time post-diagnosis and cancer stage, level of depressive symptoms at baseline, the presence of other comorbidities) and investigate whether the distribution of these variables is similar across the studies included in pairwise comparisons (see “Heterogeneity and inconsistency assessments” section for further details) [34].

### Statistical analysis

We will use Stata 15 to carry out all quantitative analysis.

#### Study and network characteristics

We will present study characteristics and descriptive statistics on important clinical and methodological variables for all included RCTs (e.g. publication year, age, sex breakdown, settings, cancer type and severity, stage of treatment). We will generate network diagrams to illustrate the amount of evidence for each outcome and the properties of the RCTs contributing to each outcome [34]. The size of each node in the diagrams will represent the number of participants in the intervention/comparator group, the edge width of the node will represent the number of RCTs involving a given intervention/comparator group while lines connecting the nodes will signify the intervention/comparison groups that have been directly compared in the available RCTs [23].

#### Pairwise meta-analysis

We will perform random-effects pairwise meta-analyses when head-to-head data are available. This will allow us to examine whether study characteristics are comparable across the RCTs that inform each direct comparison (see “Heterogeneity and inconsistency assessments” section), explore the impact of any potential outliers [63] and identify differences in estimated effects from the NMA that may be due to correlations between outcomes [28]. For each pairwise analyses, we will report SMD or odds ratios, both with associated 95% confidence intervals, for continuous and binary outcomes, respectively [64, 65].

#### Heterogeneity and inconsistency assessments

We will explore the impact of effect modifiers within and across comparisons, which may lead to heterogeneity and inconsistency respectfully, using both local and global measures [34]. Specifically, we will use the design-by-treatment interaction model to assess inconsistency in networks as a whole [61]. If we find evidence of inconsistency, we will contrast direct evidence with indirect evidence from specific loops (loop specific) and from the entire network (node specific) to detect pairwise comparisons or loops of evidence that may be introducing inconsistency in the network locally [34, 61]. We will graphically present effect sizes using forest plots to explore the possibility of statistical heterogeneity. Furthermore, we will quantify statistical heterogeneity and statistical inconsistency for each pairwise meta-analysis using *I*^2^.

#### Network meta-analysis

We will carry out a frequentist random-effects multivariate network meta-analysis to synthesise all evidence for each outcome and obtain a comprehensive ranking of all intervention groups for the primary outcomes. To this end, we will use the commands *network meta* and *mvmeta* (which underpins the first command) in Stata 15 [64]. These commands use a Newton-Raphson procedure to carry out a restricted maximum likelihood (REML) multivariate meta-analysis, which accounts for within- and between-study correlations. Our analysis will include all available interventions types and comparator groups, as described and grouped above (i.e. pharmacology interventions, psychotherapy interventions). If sufficient data are available, we will perform a second analysis that separates the various groupings by subtype (e.g. type of psychotherapy). We will use rankograms and surface under the cumulative ranking (SUCRA) curves to rank intervention groups and visually present the uncertainty in ranking probabilities [34]. An intervention/intervention groups’ SUCRA value corresponds to the ratio of the area under the cumulative ranking curve to the entire area in the plot. As such, it refers to the percentage of effectiveness/acceptability of an intervention/intervention group relative to a hypothetical intervention that would be rated the best without any uncertainty [23].

#### Sensitivity analysis

We will perform sensitivity analyses to examine the robustness of our findings with regard to the primary outcomes by carrying out subgroup analysis for the following, provided sufficient data are available:

1. Studies with different levels of bias (i.e. low, of some concern, high)
2. Studies of patients who meet the criteria for depression on a diagnostic interview and patients who score above the cut-off on a validated measure of depression
3. Studies that targeted depression as the primary outcome and those that did not and/or included depression as one of a number of outcomes
4. Studies of patient groups at different stages of treatment (i.e. patients who are pretreatment, on active treatment or post-treatment)
5. Studies of patients with advanced/incurable cancers or receiving palliative care (i.e. advanced cancer vs. early-stage disease)
6. Studies of patients with different cancer types (e.g. breast cancer vs. other cancers)
7. Studies that include patients with a premorbid history of depression and those who do not

#### Bias assessments

We will consider the likelihood that studies were conducted and not published and the comprehensiveness of our search strategy in evaluating the possibility of publication bias. We will use funnel plots and Egger’s test to evaluate publication bias and the influence of smaller studies in pairwise comparisons [34, 61]. Furthermore, we will assess asymmetry on comparison-adjusted funnel plots, to examine possible associations between study size and study effect [23]. Comparison-adjusted funnel plots graph the inverted standard error of the effect size on one axis to an adjusted effect size, which comprises the difference between a study estimate and their direct meta-analysis mean effect, and can be used to examine whether results vary depending on trial precision in NMAs [34, 61]. Finally, we will carry out a network-meta regression to determine associations between study size and effect size [66].

## Discussion

Despite the increased risk of depressive symptoms among patients with cancer compared to the general population, many patients with cancer who experience depression do not receive interventions for depression [2, 67]. Increasing recognition of the urgent need to offer greater supports to patients and reduce distress has led to a greater push for depression screening and holistic needs assessments for patients with cancer [67, 68]. However, screening will only benefit patients if it leads to effective treatments and supports [8]. Furthermore, if effective interventions are perceived to be lacking, patients and healthcare professionals may be hesitant to discuss symptoms of depression [8]. The findings from this NMA will provide the best available evidence for managing depression among patients with cancer and provide treatment rankings in terms of both efficacy and acceptability. In addition, our findings will give insight into whether diverse management approaches differentially impact on quality of life and mortality, as well as rates of adverse events associated with treatments. Such information will help to inform clinical guidelines and be of key importance to healthcare professionals and patients, helping them to make informed and evidence-based decisions about what treatment approach, or combination of approaches, is likely to benefit them most. This information will also be valuable for policy-makers, funders, and health service providers in informing the development of psycho-oncological services and service configuration. Furthermore, by providing greater clarity on the treatments that are available and effective, findings from this NMA could provide evidence to rigorously supplement the evaluation of depression screening programmes.

Our review will help to identify existing gaps in the literature, by highlighting available interventions for managing depression among patients with cancer for which there is currently insufficient evidence because of a lack of available research. Similarly, by examining the consistency of the results across different types of cancer and stages of cancer treatment, our review will identify when existing data relating to certain cancer cohorts, such as rare cancers, are lacking. It is probable that our review and our ability to draw conclusions regarding the best available treatments for specific cancer cohorts will be limited by the data that are available, and this will likely be a significant limitation of the review. Nevertheless, identifying the gaps in the existing evidence base will also be of value.

Another key limitation of this NMA is that our results will not capture patient preferences and choice beyond treatment acceptability, which is our second primary outcome. It is probable that many trials do not account for patient preferences, and, in carrying out the statistical analysis, we can only use the outcome data that is presented. However, to consider patient preference and choice beyond crude treatment acceptability, we will also be carrying out a qualitative study as an additional part of this project to discuss our findings with key patient and healthcare provider stakeholders. This qualitative study will explore stakeholders’ views of the findings, their experiences of and access to these treatments and their views on applying these findings in clinical practice. We hope that insights from the qualitative study will help to bridge the gap between evidence-based medicine and the experiences of patients with cancer and healthcare professionals in the “real world”.

## Supplementary Information

**Additional file 1..** PRISMA-P 2015 Checklist: ENHANCE Project.

**Additional file 2: Table 1.** ENHANCE project Public and Patient Involvement: GRIPP2 short form.

**Additional file 3..** Sample Search Strategy for Systematic Reviews and Meta-analyses. Sample Search Strategy for Randomized Controlled Trials.

## Data Availability

Not applicable

## Abbreviations

CINeMA: Confidence in NEtwork Meta-Analysis
CAM: Complementary and alternative medicine
DSM5: *Diagnostic and Statistical Manual of Mental Disorders, 5th Edition*
GRADE: Grading of Recommendations Assessment, Development and Evaluation framework
GRIPP2-SF: Guidance for Reporting Involvement of Patients and the Public 2-short form
HAM-D: Hamilton Depression Rating Scale
HRQoL: Health-related quality of life
NMA: Network meta-analysis
PICO: Patient, intervention, comparison, and outcome
PPI: Public and patient involvement
RCTs: Randomised controlled trials
REML: Restricted maximum likelihood
RoB: Risk of bias
SDs: Standard deviations
SMD: Standardised mean difference
SUCRA: Surface under the cumulative ranking
TIDiER: Template for intervention description and replication checklist
TAU: Treatment as usual
WHO ICTRP: World Health Organization International Clinical Trials Registry Platform
