# Supplementary material for: Effective maNagement of depression among patients witH cANCEr (ENHANCE): a protocol for a hybrid systematic review and network meta-analysis of randomised controlled trials of interventions for depressive symptoms
Source: Syst Rev. 2022 Nov 12;11:239. doi: 10.1186/s13643-022-02107-y (PMC9655794; doi:10.1186/s13643-022-02107-y)
Supplement: Supplementary file 2 — Additional file 2: Table 1. ENHANCE project Public and Patient Involvement: GRIPP2 short form. [file 13643_2022_2107_MOESM2_ESM.docx]

Table 1. ENHANCE project Public and Patient Involvement: GRIPP2 short form

| Section and topic: | Report |
| --- | --- |
| 1: Aim.  Report the aim of PPI in the study. | The aim of PPI in this study is to refine the focus of the research questions, ensuring they are operationalised in a way that is appropriate and relevant to patients with cancer who experience depression, and to assist with the interpretation and dissemination of findings from the network meta-analysis (NMA). |
| 2: Methods.  Provide a clear description of the methods used for PPI in the study. | At the outset of the project, a PPI panel comprising of two experts by experience was recruited to the steering committee, which will meet four times per year. Prior to the first steering group meeting, the ENHANCE PPI panel members took part in a training session, during which they were given an overview of the research methods pertinent to the project and an outline of the proposed study protocol was discussed. The PPI panel gave their views on the PICO criteria for study eligibility and these were incorporated into the draft manuscript for this protocol and/or flagged for further discussion at the steering group meeting. Through these discussions, PPI input helped to refine decisions on study exclusion and inclusion criteria.  While the ENHANCE PPI panel did not have input into the development of the research questions, the funding application for this project was reviewed by the funder’s PPI panel, who played a key role in selecting this project for funding. Feedback from this panel, which was comprised of people with experience of cancer, indicated that they deemed the focus of the study to be important and likely to have a beneficial impact on cancer care. Feedback from these PPI reviewers, which centred on the dissemination of findings to achieve maximum impact, was also integrated into the final project proposal.  Going forward, the ENHANCE PPI panel will continue to contribute to the project by helping with the interpretation of findings from the NMA and considering their implications for practice. In addition, they will help with the dissemination of the completed NMA, by contributing to lay reports and summaries, presentations and edits of the final paper.  The ENHANCE PPI panel are named as co-authors on this protocol. |
| 3: Study results.  Outcomes - Report the results of PPI in the study, including both positive and negative outcomes. | Results are pending since this only relates to the study protocol; however, outcomes of PPI contributions to date include:   - Modification of study PICOs; for example, the nature of included interventions (e.g., to include complementary and alternative medicine interventions).   Results will be reported in subsequent publications. |
| 4: Discussion and conclusions.  Outcomes—Comment on the extent to which PPI influenced the study overall. Describe positive and negative effects | Not applicable since this is a protocol. Discussion and conclusions will be reported in subsequent publications. |
| 5: Reflections/critical perspective.  Comment critically on the study, reflecting on the things that went well and those that did not, so others can learn from this experience | Not applicable since this is a protocol. Reflections on PPI involvement will be reported in subsequent publications. |

ENHANCE = Effective maNagement of depression among patients witH cANCEr; GRIPP2-SF: Guidance for Reporting Involvement of Patients and the Public 2 - Short Form; PPI = patient and public involvement; PICO = Participants, Intervention, Comparison, Outcome
