# Supplementary material for: Effective maNagement of depression among patients witH cANCEr (ENHANCE): a protocol for a hybrid systematic review and network meta-analysis of randomised controlled trials of interventions for depressive symptoms
Source: Syst Rev. 2022 Nov 12;11:239. doi: 10.1186/s13643-022-02107-y (PMC9655794; doi:10.1186/s13643-022-02107-y)
Supplement: Supplementary file 3 — Additional file 3.. Sample Search Strategy for Systematic Reviews and Meta-analyses. Sample Search Strategy for Randomized Controlled Trials. [file 13643_2022_2107_MOESM3_ESM.docx]

# Additional File 3

Sample Search Strategy for Systematic Reviews and Meta-analyses

|  | **Ovid MEDLINE(R) ALL** |
| --- | --- |
| 1 | exp Cancer/ OR exp Neoplasm/ OR cancer$.mp. OR neoplasm$.mp. |
| 2 | Depression/ OR depression.mp. OR exp Depressive Disorder/ OR Mood Disorders/ OR dysthymi$.mp. OR (depressi$ adj3 disorder$).mp. OR (depressi$ adj3 symptom$).mp. OR mood disorder$.tw OR affective disorder$.mp. |
| 3 | randomized controlled trial/ OR randomized controlled trial.pt. OR controlled clinical trial.pt. OR controlled clinical study/ OR random*.ti,ab. OR randomization/ OR double blind procedure/ OR ((double or single or doubly or singly) adj1 (blind or blinded or blindly)).ti,ab. OR placebo.ti,ab. OR trial$.ti,ab. OR control group.ti,ab. |
| 4 | Meta-analysis.mp. OR meta-analysis.pt. OR meta-analysis.sh. OR (meta adj1 analys*) OR (systematic adj3 review$).mp. OR (systematic adj3 overview$).mp. OR (quantitative adj3 review$).mp. OR (quantitative adj3 overview$).mp. OR (quantitative adj3 synthesis).mp. OR (methodologic adj3 review$).mp. OR (methodologic adj3 overview$).mp. OR (integrative research review$ OR research integration).mp. OR review.pt. |
| 5 | 1 AND 2 AND 3 AND 4 |
| 6 | Limit to HUMANS |

Sample Search Strategy for Randomized Controlled Trials

|  | **Ovid MEDLINE(R) ALL** |
| --- | --- |
| 1 | exp Cancer/ OR exp Neoplasm/ OR cancer$.mp. OR neoplasm$.mp. |
| 2 | Depression/ OR depression.mp. OR exp Depressive Disorder/ OR  Mood Disorders/ OR dysthymi$.mp. OR (depressi$ adj3 disorder$).mp. OR (depressi$ adj3 symptom$).mp. OR mood disorder$.tw OR affective disorder$.mp. |
| 3 | 1 AND 2 |
| 4 | randomized controlled trial/ OR randomized controlled trial.pt. OR controlled clinical trial.pt. OR random*.ti,ab. OR randomization/ OR double blind procedure/ OR ((double or single or doubly or singly) adj1 (blind or blinded or blindly)).ti,ab. OR placebo.ti,ab. OR trial$.ti,ab. OR  control group.ti,ab. |
| 5 | 3 AND 4 |
| 6 | (comment or editorial or review or letter).pt. |
| 7 | 5 NOT 6 |
| 8 | (animals NOT humans).sh. |
| 9 | 7 NOT 8 |
| 10 | limit 9 to yr="2016 - 2021"* |

* We anticipate that the time-period for updated RCT searches will be within the last 3 - 5 years; however, the range will be determined by how recent the available systematic reviews are.
